# Network Pharmacology and Inflammatory Microenvironment Strategy Approach to Finding the Potential Target of *Siraitia grosvenorii* (Luo Han Guo) for Glioblastoma

**DOI:** 10.3389/fgene.2021.799799

**Published:** 2021-12-20

**Authors:** Juan Li, De Bi, Xin Zhang, Yunpeng Cao, Kun Lv, Lan Jiang

**Affiliations:** ^1^ Key Laboratory of Non-coding RNA Transformation Research of Anhui Higher Education Institution, Yijishan Hospital of Wannan Medical College, Wuhu, China; ^2^ Suzhou Polytechnic Institute of Agriculture, Suzhou, China; ^3^ Wuhan Botanical Garden, Chinese Academy of Sciences, Wuhan, China; ^4^ Central Laboratory, Yijishan Hospital of Wannan Medical College, Wuhu, China

**Keywords:** *Siraitia grosvenorii*, CCL5, glioblastoma, in silico, network pharmacology

## Abstract

**Background:** Glioblastoma (GBM) is the most common and aggressive primary intracranial tumor of the central nervous system, and the prognosis of GBM remains a challenge using the standard methods of treatment—TMZ, radiation, and surgical resection. Traditional Chinese medicine (TCM) is a helpful complementary and alternative medicine. However, there are relatively few studies on TCM for GBM.

**Purpose:** We aimed to find the connection between TCM and anti-GBM.

**Study design:** Network pharmacology and inflammatory microenvironment strategy were used to predict *Siraitia grosvenorii* (Luo Han Guo) target for treating glioblastoma.

**Methods:** We mainly used network pharmacology and bioinformatics.

**Results:** CCL5 was significantly highly expressed in GBM with poor prognostics. Uni-cox and randomForest were used to determine that CCL5 was especially a biomarker in GBM. CCL5 was also the target for SG and TMZ. The active ingredient of Luo Han Guo — squalene and CCL5 —showed high binding efficiency. CCL5, a chemotactic ligand, was enriched and positively correlated in eosinophils. CCL5 was also the target of Luo Han Guo, and its effective active integrate compound –— squalene — might act on CCL5.

**Conclusion:** SG might be a new complementary therapy of the same medicine and food, working on the target CCL5 and playing an anti-GBM effect. CCL5 might affect the immune microenvironment of GBM.

## Introduction

Glioblastoma (GBM) is the most common and aggressive primary intracranial tumor of the central nervous system (Barthel et al., 2019; Miller et al., 2019). Most of them are induced by genetic mutations of high penetrance genes related to rare syndromes, mainly manifested as increased intracranial pressure, neurocognitive dysfunction, and seizures, resulting in central nervous system damage and endangering the lives of patients (Zanders et al., 2019). The standard treatment for GBM is surgery, drug therapy, and radiation therapy, and the median survival time of patients is only 15 months (Kumar et al., 2019). With the changes in eating habits, living environment, and work pressure, the incidence of GBM is increasing and getting younger. Surgical resection combined with postoperative radiotherapy, chemotherapy, and immunotherapy will inevitably damage the body’s normal function and cause adverse reactions. Multi-drug resistance, especially temozolomide (TMZ), leads to frequent GBM recurrences, which is a challenge in treating GBM, and its underlying molecular mechanism is still unclear (Yin et al., 2019). Since the blood-brain barrier (BBB) can prevent the accumulation of charged or macromolecules in the tumor microenvironment at a physically relevant concentration, thereby exerting an oncolytic effect, the content of TMZ in the brain is only 40% percent of the content in the blood, and new component pharmacological methods must be developed to enhance the curative effect of the current treatment, prolong the median survival time of the patient to exceed the median survival time of 15 months (Kumar et al., 2019).

The plants of traditional Chinese medicine (TCM) were used for the treatment of various cancers (Dai et al., 2016), such as GBM (Wang et al., 2019). The use of TCM to promote health and adjuvant therapy is becoming increasingly popular worldwide (Khan and Tania, 2020). The active components of *Salvia miltiorrhiza* can inhibit the proliferation of U87 cells, induce apoptosis, and enhance the efficacy of TMZ (Wang et al., 2019). *Lycium chinense* can up-regulate CD3+T, CD8+T, and TNF-α, inhibit the proliferation of mouse C6 cells, and up-regulate CD4^+^CD5+T cells to prolong survival and regulate the BBB (Wang et al., 2019). Magnolol inhibits the migration and proliferation of GBM cells through the JAK-STAT3 signal pathway, mainly by inhibiting the production of GBM stem cell-like cells (Fan et al., 2019). However, the clinical application value of TCM in the treatment of GBM has not been promoted, and more molecular mechanism studies are needed to verify it. Therefore, our research aims to provide new potential for the treatment of GBM with a medicinal plant.

The TCM *Siraitia grosvenori* (SG) is a perennial herbaceous plant of the Cucurbitaceae family with huge resource reserves and native to southern China, also known as monk fruit and Luo Han Guo, which is a medicinal food homologous species granted by the China Food and Drug Administration with significant clinical effects (Xia et al., 2018). Mogroside has an excellent biological development, which can inhibit the excessive activation of Signal Transducer and Activator of Transcription 3 (STAT3) and promote tumor cell apoptosis (Liu et al., 2018), and targeting STAT3 can improve tumor progression and anticancer immunity response (Lee et al., 2019); reversing emergency medical technician (EMT) and destroying the cytoskeleton to inhibit hyperglycemia-induced lung cancer cell metastasis (Guan et al., 2019). Mogroside IIV and IIIV activate AMP-activated protein kinase (AMPK) and produce anti-hyperglycemic and anti-lipid properties in the body (Abdel-Hamid et al., 2020); mogroside V can cross the BBB and affect schizophrenia-like behavior (Ju et al., 2020) and can also exert neuroprotective activity (Xia et al., 2013); mogroside IVe may be potentially used as a bioactive phytochemical supplement for the treatment of colorectal cancer and laryngeal cancer (Liu et al., 2016). Monk fruit also has other pharmacological effects, such as up-regulating Sirtuin 1 (SIRT1) to reduce oxidative stress and alleviate the decline in oocyte quality during *in vitro* aging (Nie et al., 2019).

**FIGURE 1:**
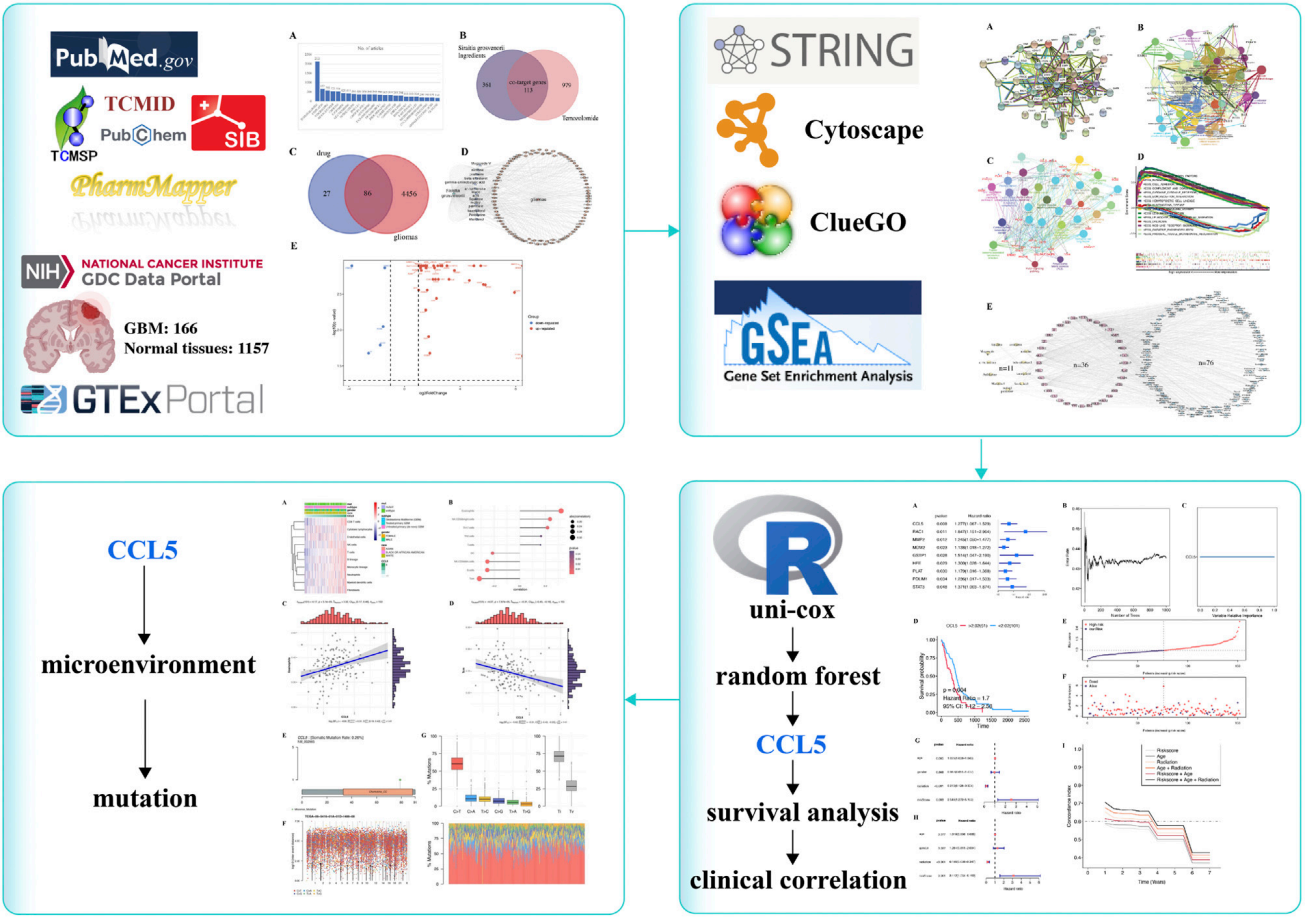
Graphical abstract. The new flowchart explains the potential target of *Siraitia grosvenorii* (Luo Han Guo) for glioblastoma.

Network pharmacology is based on the high-throughput multi-omics data analysis to clarify the mechanism of multi-component/multi-target/multiple action pathways in medicinal plants (Hopkins, 2008). The newly network pharmacology analysis was employed to integrate active compounds, targets and pathways prediction, and network analysis which may provide novel insights into the therapeutic effects and molecular mechanisms of SG in the treatment for GBM (Abdel-Hamid et al., 2020). Then, we offered a new flowchart to explain the potential target of *Siraitia grosvenorii* (Luo Han Guo) for GBM (Figure 1).

## Materials and Methods

### The Integration of SG-TMZ-GBM (*Siraitia grosvenori* - Temozolomide - Glioblastoma) Targets

Through PubMed database (https://pubmed.ncbi.nlm.nih.gov) text mining, we selected the most effective clinical drug in the treatment of GBM. Based on the TCMSP (Ru et al., 2014) database (blood-brain barrier (BBB) ≥ 0.3, drug-like (DL) ≥ 0.18, oral bioavailability (OB) ≥ 30%), and TCMID (Huang et al., 2018), we collected the active ingredients and targets in monk fruit. Then, we used the chemical components to obtain the structure files by the PubChem Compound database (Kim et al., 2019) and uploaded the structure files to predict the targets across the PharmMapper (Wang et al., 2017) and Swiss Target Prediction (Gfeller et al., 2014). A Venn diagram (http://bioinformatics.psb.ugent.be/webtools/Venn/) was drawn for visualizing the SG-TMZ interacting targets. Gliomas-related targets were predicted by OMIM (Amberger et al., 2015), DrugBank (Wishart et al., 2018), and PubMed. Then, taking the intersection with the prediction targets of SG-TMZ, which is named Siraitia grosvenori - temozolomide – gene (SG-TMZ-G).

We downloaded GBM’s transcriptomic and clinical data and normal brain tissues from XENA TCGA and GTEx (https://xena.ucsc.edu/public/). Differentially expressed GBMs (DE-GBMs) were computed by limma (Smyth, 2005) with |logFoldChange (logFC)| > 2 and q-value < 0.05 as previously reported (Jiang et al., 2020a; Jiang et al., 2020b). Subsequently, common GBM-related targets were integrated between SG-TMZ-G. A volcano plot was used to show the distribution of SG-TMZ-GBM (SG-TMZ-glioblastoma).

### Functional Analysis and Network Construction of SG-TMZ-GBM

STRING v11.5 was used to construct a protein-protein interaction (PPI) network, scores >0.70 were considered to have high confidence (Szklarczyk et al., 2021). Functional analyses of the gene ontology (GO), the Kyoto Encyclopedia of Genes and Genomes (KEGG) were performed by ClueGO plug-in (Bindea et al., 2009) in Cytoscape v3.8.2 (Reimand et al., 2019) with q-value ≤ 0.001. The compound-target-pathway network was built by Cytoscape (Reimand et al., 2019).

### The Determination of the Key SG-TMZ-GBMs

Hazard ratios (HR) were applied using univariable Cox (uni-cox) regression analysis (p-value < 0.05). We then detected the key SG-TMZ-GBMs by “survival” and “survminer” package (Jiang et al., 2020b). Random forest was calculated by randomSurvivalForest to rank the importance of survival-related SG-TMZ-GBMs, with a relative importance >0.7 as the final feature (Liu et al., 2021). Survival analysis was built with the best cutoff value (Liu et al., 2021), the Kaplan-Meier method was used to draw survival curves, and the log-rank test was used to evaluate differences. A scatter plot of C-C Motif Chemokine Ligand 5 (CCL5) expression and survival time in GBM patients were drawn by ggrisk (Jiang et al., 2020b). The forest plot was used for performing uni-cox and multiple cox (multi-cox) regression analysis (Jiang et al., 2020b). We also used the receiver operating characteristic curve (ROC), concordance index (c-index) to evaluate the multi-clinical prognostic performance (Longato et al., 2020).

## Inflammatory Microenvironment and Mutation Analysis

The microenvironment cell population-counter method was chosen to evaluate the association between CCL5 and immune cell populations (Petitprez et al., 2020). We used immune cells markers and GBM transcriptome data to validate the strong correlation between CCL5 and 24 immune cells markers (Bindea et al., 2013). Gene mutations of GBM expression by “maftools” package (Mayakonda et al., 2018). CCL5 protein expression was detected by immunohistochemistry from the HPA database (https://www.proteinatlas.org/ENSG00000271503-CCL5/pathology/glioma#).

## Results

### SG-TMZ-GBM Detection

**FIGURE 2:**
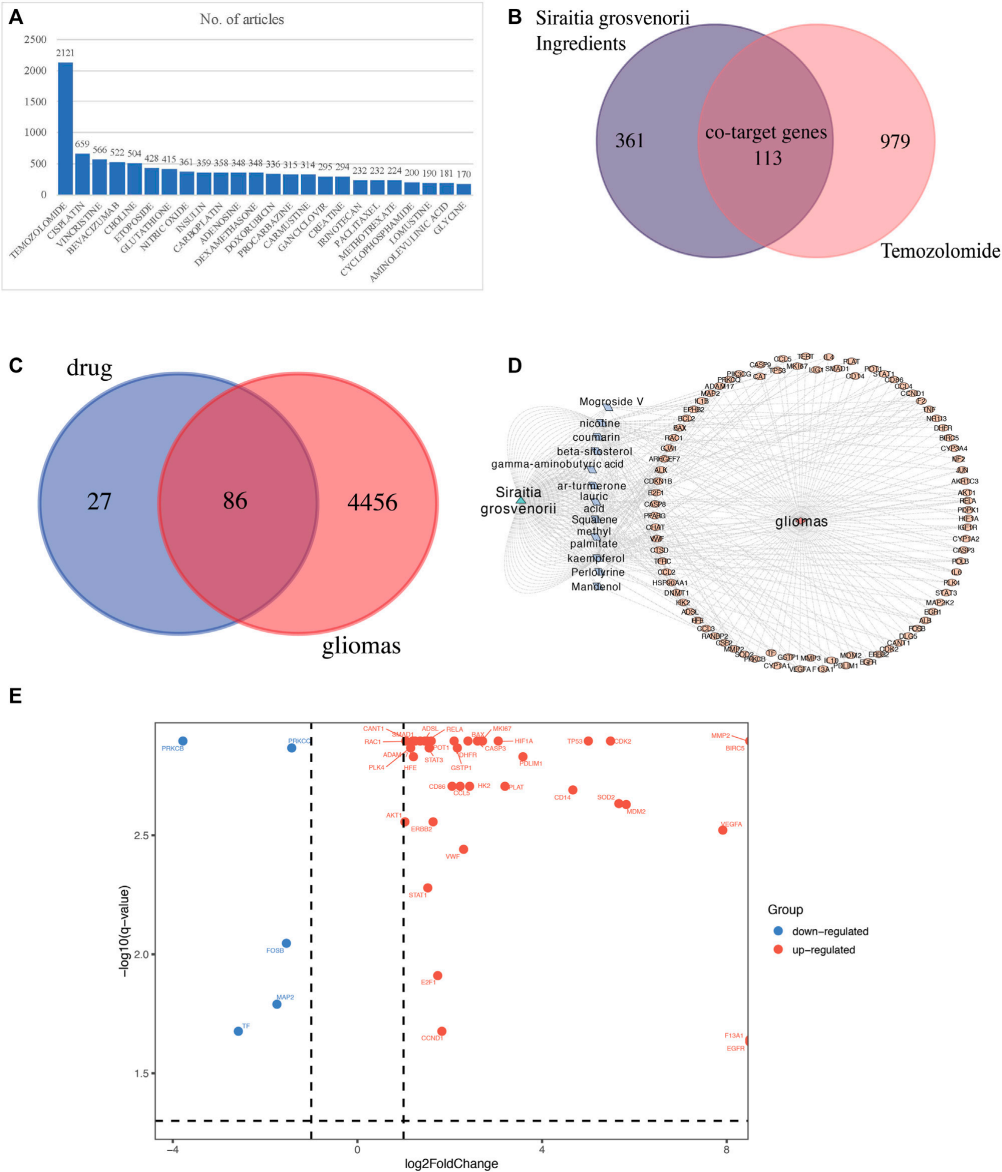
SG-TMZ-GBM detection. **(A)** The analysis result on the clinical drug in treating gliomas in PubMed database (updated by 2021-8-10); **(B)** co-target genes predicted between the ingredients of *Siraitia grosvenorii* and temozolomide; **(C)** co-target genes predicted between the ingredients of *Siraitia grosvenorii* -temozolomide and gliomas; **(D)** co-target genes predicted network between the ingredients of *Siraitia grosvenorii* and gliomas, a triangle means *Siraitia grosvenorii*, a diamond means the 12 ingredients of *Siraitia grosvenorii*, an oval means co-target genes in gliomas; **(E)** volcano plot for differentially expressed target genes.

PubMed text mining showed 2121 literature reports on the treatment of GBM with TMZ (Figure 2A). Through the text data mining of the Therapeutic Target Database (TTD) database and PubMed published articles, we identified 1092 target genes for treating GBM with TMZ.

We obtained 12 chemical compositions in SG by TCMSP and TCMID, and gained the compound structure by PubChem, and predicted the 474 target genes of SG by the Swiss Target Prediction and PharmMapper. A total of 113 SG-TMZ targets were found by taking the intersection (Figure 2B). We further discovered 4542 target genes related to gliomas through PALM-IST, filtered 86 target genes as SG-TMZ-G (Figure 2C), and drew a network diagram (Figure 2D). For example, IL6 was a co-target gene in the gamma-aminobutyric acid, lauric acid, and methyl palmitate of SG and GBM; CCL5 was a co-target gene in squalene. According to the cutoff log2FoldChange> 2 and q-value <0.05, we screened the interaction of differentially expressed genes (DEGs) in GBM-normal brain tissues and SG-TMZ-G, Volcano plot for 42 SG-TMZ-GBM targets were detected for the following research (Figure 2E).

### Luo Han Guo Compound-Target-Disease Interaction Network and Functional Enrichment Analysis

**FIGURE 3:**
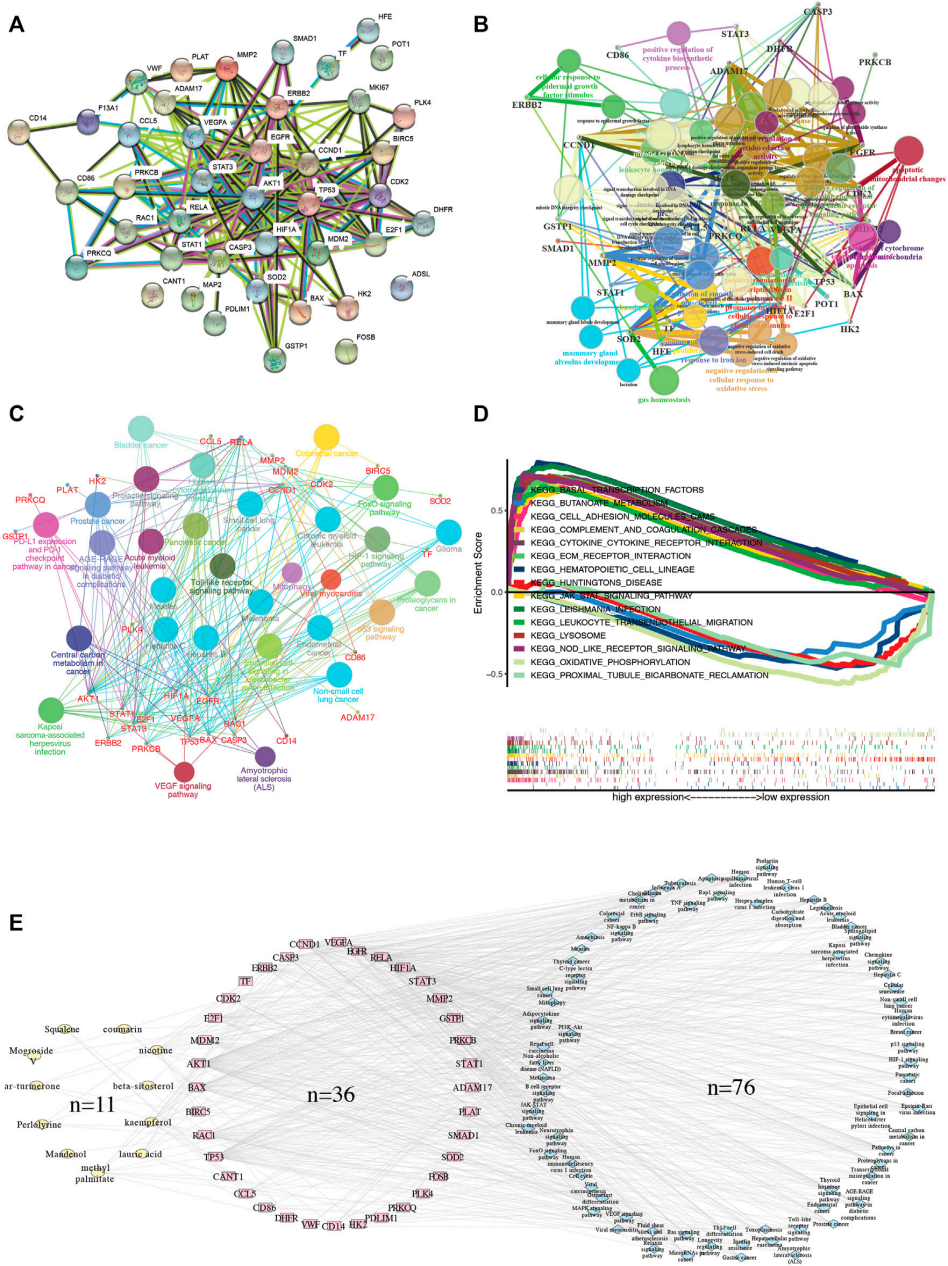
Targeted gene functional analysis. **(A)** PPI network analysis; **(B)** GO enrichment analysis; **(C)** KEGG pathway enrichment analysis; **(D)** the study of GSEA KEGG enrichment plot. **(E)** Compound-target-KEGG pathway network. The network was generated by Cytoscape 3.8.2. Yellow circles represent 11 ingredient targets from *Siraitia grosvenorii*. Red circles represent 36 common targets between ingredient targets from *Siraitia grosvenorii* and GBM significant targets. Blue circles represent 76 KEGG pathways.

We imported 42 SG-TMZ-GBMs into the STRING database to construct a protein-protein interaction (PPI) network, the primary connection in the network which might have pharmacological effects in GBM. In addition, the four targets, including Protection of Telomeres 1 (POT1), Adenylosuccinate Lyase (ADSL), FosB Proto-Oncogene, AP-1 Transcription Factor Subunit (FOSB), and Calcium Activated Nucleotidase 1 (CANT1), did not interact with other targets (Figure 3A). Tumor Protein P53 (TP53) and MDM2 Proto-Oncogene (MDM2), Cyclin Dependent kinase 2 (CDK2) and Cyclin D1 (CCND1) (scores >0.70) were considered to have high confidence. We further explore the correlation between 42 SG-TMZ-GBMs and glioblastoma by GO (Figure 3B), KEGG (Figure 3C), and GSEA (Figure 3D) enrichment analyses. We discovered that 54 significant GO enrichment results, such as “lactation,” “response to iron ion,” “apoptotic mitochondrial changes,” CCL5 was enriched in “human cytomegalovirus infection,” “toll-like receptor signaling pathway” and “epithelial cell signaling in *helicobacter pylori* infection,” Vascular Endothelial Growth Factor A (VEGFA), Rac Family Small GTPase 1 (RAC1), Protein kinase C Beta (PRKCB), and AKT Serine/Threonine kinase 1 (AKT1) were enriched in Vascular Endothelial Growth Factor (VEGF) signaling pathway (Figure 3B); pathway analysis revealed that SG-TMZ-GBMs were associated with cancer-related pathway, including glioma, non-small cell lung cancer, pancreatic cancer, and thyroid cancer, AKT1, BCL2 Associated X, Apoptosis Regulator (BAX), CCND1, E2F Transcription Factor 1 (E2F1), MDM2, PRKCB, and TP53 were enriched in “glioma” pathway, suggesting Luo Han Guo may play a role in cancer treatment; PRKCB, RELA Proto-Oncogene, NF-KB Subunit (RELA), STAT3, and VEGFA were enriched in “AGE-RAGE signaling pathway” and “HIF-1 signaling pathway” might be related in the inflammation-related diseases (Figure 3C). The GSEA KEGG enrichment analysis is shown in Figure 3D, and we found the top three significantly activated KEGG pathways were “KEGG hematopoietic cell lineage”, “KEGG leishmania infection”, and “KEGG nod like receptor signaling pathway”. A compound-target-pathway network was established based on the target recognition and pathway analysis, with nodes mapping compounds, targets, or pathways, and indicated interactions by Cytoscape (Figure 3E).

### The Determination of the Key SG-TMZ-GBMs

**FIGURE 4:**
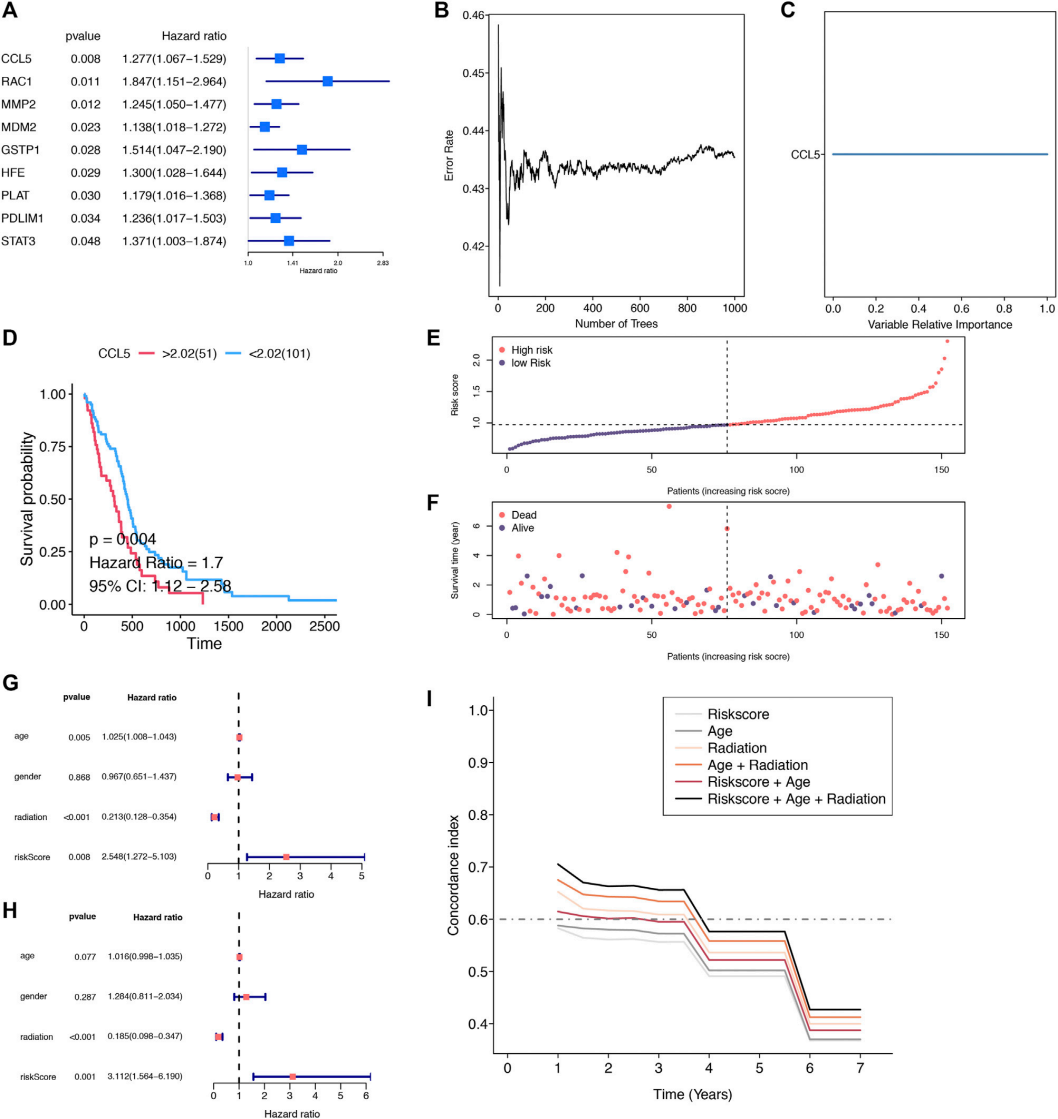
The determination of the key SG-TMZ-GBMs. **(A)** Uni-cox analysis; **(B)** error tree of randomForest; **(C)** variable relative importance of randomForest; **(D)** survival analysis; **(E)** the curve of risk score; **(F)** survival status by ggrisk; **(G)** uni-cox with clinical information; **(H)** multi-cox with clinical information; and **(I)** ROC-concordance index.

Uni-cox analysis revealed that 9 SG-TMZ-GBMs were determined as the significant survival-related risk genes. CCL5 was the most significant gene (p-value = 0.008) (Figure 4A). We found CCL5 was the key SG-TMZ-GBM (importance = 1) by random forest calculation (Figures 4B,C) and the survival analysis with p-value = 0.004 (Figure 4D). To further explore the effect of CCL5 on tshe GBM prognosis, a scatter plot of CCL5 expression and survival time in GBM patients was created (Figures 4E,F). Uni-cox and multi-cox regression analysis revealed that radiation (p-value < 0.001) and risk score (p-value = 0.008) were independent risk factors for overall survival analysis (Figures 4G,H). ROC c-index analysis illustrated that risk score + age + radiation, age + radiation, and radiation were the top three (Figure 4I).

## Inflammatory Microenvironment and Mutation Analysis

**FIGURE 5:**
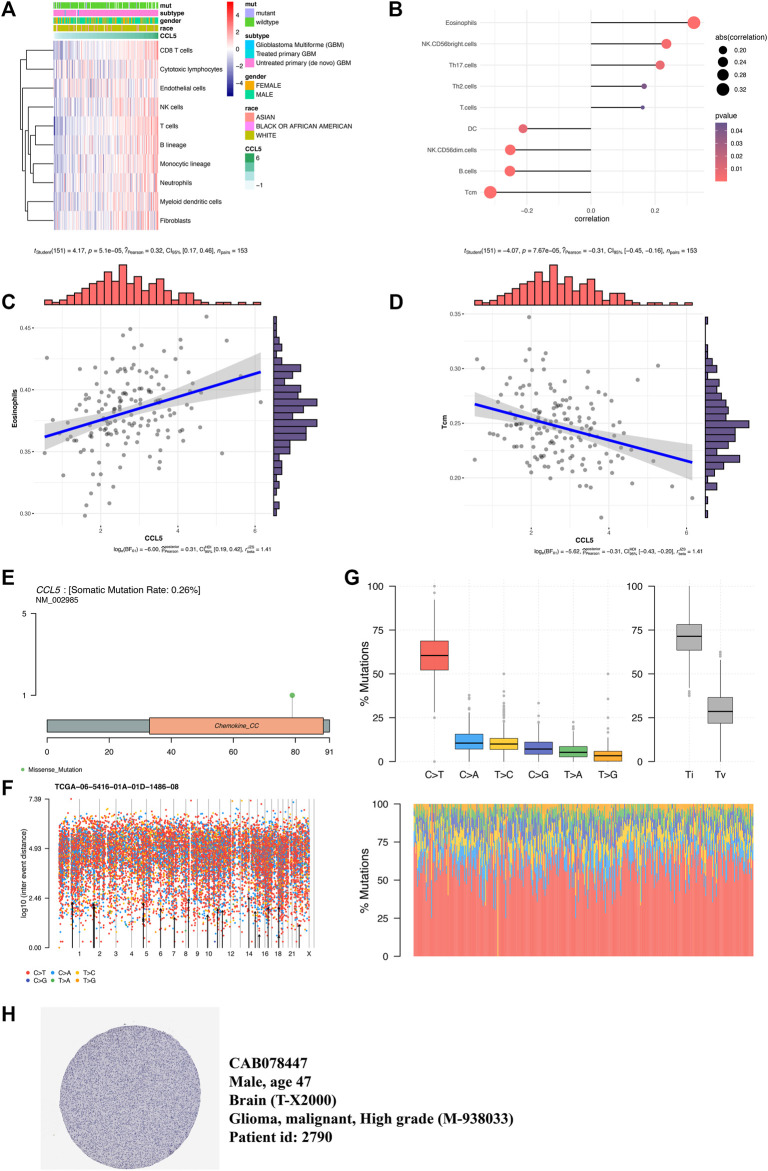
Inflammatory microenvironment and mutation analysis. **(A)** Association between CCL5 expression and 10 immune cell populations in GBM. **(B)** The association between CCL5 expression and 24 immune cell markers in GBM. **(C)** A scatter plot of the positive correlation between CCL5 expression and eosinophils. **(D)** A scatter plot of the negative correlation between CCL5 expression and Tcm. **(E)** The lollipop map shows the mutation distribution and protein domain of CCL5 with somatic mutation. **(F)** The rainfall map of TCGA-AC-A23H-01A-11D-A159-09 in the GBM sample. **(G)** The transition and crosscut graphs show the distribution of SNV in GBM with six transition and crosscut events. The stacked bar graph **(bottom)** shows the mutation spectrum distribution of each sample. **(H)** CCL5 protein expression can be detected by immunohistochemistry from the HPA database.

The microenvironment cell population-counter method evaluated the association between CCL5 and 10 immune cell populations from transcriptomic data. A strong correlation between CCL5 and CD8 T cells, T cells, B lineage, and fibroblasts were seen (Figure 5A). Then we further found the significant correlation (p-value < 0.05) between CCL5 and 9 of 24 immune cells markers (Figure 5B), such as the positive correlation in eosinophils (Figure 5C) and the negative correlation in Tcm (Figure 5D). In addition, exploring somatic mutations is helpful to understand the occurrence and development of GBM. The lollipop map shows the mutation distribution and protein domain of CCL5 with somatic mutation (Figure 5E). The distribution of the mutation spectrum of GBM samples can also be identified by a rainfall map (Figure 5F). The transition plot classified single nuclear variants into six categories (Figure 5G). Among them, the C > T mutation accounted for more than 50% of the total mutations. Furthermore, CCL5 protein expression can be detected by immunohistochemistry from the HPA database (Figure 5H).

## Discussion

GBM is the most frequent and the least treatable type of brain tumor, and the prognosis of GBM remains a challenge using the standard methods of treatment—TMZ, radiation, and surgical resection (Jiang et al., 2020b). TMZ is a novel methylating agent that demonstrated activity against recurrent GBM and is ineffective due to drug resistance (Wu et al., 2021). TCMs were considered anti-GBM auxiliary drugs, such as *Solanum nigrum L*. (Li et al., 2021), *Panax ginseng*, licorice, *Lycium barbarum*, *Salvia miltiorrhiza bunge*, *Coptis rhizoma*, and *Sophora flavescens* (Wang et al., 2019). TCM is a helpful complementary and alternative medicine, however, there are few studies on TCM for GBM (Wang et al., 2019). The anti-GBM effects of TCM extract provided the new medium for the treatment of GBM (Li et al., 2021).

We tried to find a new TCM complementary method to treat GBM and hope that through combining Chinese and Western medicine, TMZ resistance could be reversed and anti-tumor therapeutic effects could be achieved. Luo Han Guo is a TCM with the same medicine and food. The multiple compounds in Luo Han Guo not only act on the same target protein, but a single compound also acts on various target proteins and multiple pathways, which reflects the “multiple components, multiple targets, and multiple pathways” of Luo Han Guo’s synergistic effect. Luo Han Guo may work with ar-turmerone, methyl palmitate, lauric acid, beta-sitosterol, gamma-aminobutyric acid, coumarin, mogroside V, and squalene. GO functional enrichment analyses reflected that most of the active ingredients in SG might target nerve cells.

Through network pharmacology and bioinformatics analysis, we found that the CCL5 molecule is a potential target of SG, TMZ, and GBM, maybe the key to the clinical development of TMZ resistance (Figure 1). CCL5-CCR5 paracrine signaling could be an effective therapeutic strategy to improve chemotherapeutic efficacy against GBM (Zhang et al., 2021). CCL5 of glioma-associated microglia/macrophages regulates glioma migration and invasion via calcium-dependent matrix metalloproteinase 2 (Yu-Ju Wu et al., 2020). Knockdown or pharmacological inhibition of CCL5 increased the sensitivity of GBM cells treated with pericyte conditioned media to TMZ (Sprowls and Lathia, 2021). CCL5 was significantly highly expressed in GBM with poor prognostic. Uni-cox and randomForest were used to determine that CCL5 was a significantly important biomarker in GBM. CCL5 was also the target for SG and TMZ. The active ingredient of Luo Han Guo — squalene and CCL5 —show high binding efficiency. SG may be used as a new complementary therapy of the same medicine and food, acting on the target CCL5 and playing an anti-glioblastoma effect. Increasing the effective content of squalene in SG also needs further research. The radiation-related factors were the most critical in ROC c-index analysis. CCL5 plays a vital role in maintaining chemotherapy and radiation resistance.

Compared to genetically distinct syngeneic GBM models, the difference in mouse GBM models was eosinophils, reported in GBM (Khalsa and Shah, 2021). Eosinophils were associated with prognostic risk in the GBM microenvironment (Liang et al., 2020). We found that the SG-TMZ-GBM target, CCL5, a chemotactic ligand, is enriched and positively correlated in eosinophils. CCL5 is also the target of Luo Han Guo, and its effective active integrate compound – squalene—might act on CCL5, thereby affecting the immune microenvironment of GBM.

## Data Availability

The original contributions presented in the study are included in the article/Supplementary Material. Further inquiries can be directed to the corresponding author.

## Glossary

ADSL: adenylosuccinate lyase
AKT1: AKT serine/threonine kinase 1
AMPK: AMP-activated protein kinase;
BAX: BCL2 associated X, apoptosis regulator
BBB: blood-brain barrier
c-index: concordance index
CCL5: C-C motif chemokine ligand 5
CANT1: calcium activated nucleotidase 1
CDK2: CDKCyclin dependent kinase 2
CCND1: cyclin D1
DEGs: differentially expressed genes
DL: drug-like
DE-GBMs: differentially expressed GBMs
EMT: emergency medical technician
E2F1: E2F transcription factor 1
FOSB: FosB proto-oncogene, AP-1 transcription factor subunit
GBM: glioblastoma
GO: gene ontology
HR: hazard ratios
KEGG: kyoto encyclopedia of genes and Genomes
logFC: logFoldChange
MDM2: MDM2 proto-oncogene
multi-cox: multivariate cox
OB: oral bioavailability
PPI: protein-protein interaction
POT1: protection of telomeres 1
PRKCB: protein kinase C beta
RELA: RELA proto-oncogene, NF-KB subunit
RAC1: Rac family small GTPase 1
ROC: receiver operating characteristic curve
SG: *Siraitia grosvenorii*
SG-TMZ-GBM: *Siraitia grosvenorii* - temozolomide – glioblastoma
STAT3: signal transducer and activator of transcription 3
SIRT1: sirtuin 1
TP53: tumor protein P53
TCM: traditional chinese medicine
TMZ: temozolomide; uni-cox, univariable Cox
VEGFA: vascular endothelial growth factor A
VEGF: vascular endothelial growth factor
